# Qing Yan Li Ge Tang, a Chinese Herbal Formula, Induces Autophagic Cell Death through the PI3K/Akt/mTOR Pathway in Nasopharyngeal Carcinoma Cells In Vitro

**DOI:** 10.1155/2021/9925684

**Published:** 2021-11-02

**Authors:** Ching-Huey Yang, Kuo-Lung Tung, Yen-Ting Wu, Cheng Liu, Sheng-Chieh Lin, Chun-Chuan Yang, Chin-Han Wu, Hong-Yi Chang, Shih-Yi Wu, Bu-Miin Huang, Yu-Yan Lan

**Affiliations:** ^1^Department of Traditional Chinese Medicine, Kaohsiung Veterans General Hospital, Pingtung Branch, Pingtung 91245, Taiwan; ^2^Department of Dental Technology, Shu-Zen Junior College of Medicine and Management, Kaohsiung 82144, Taiwan; ^3^Department of Pathology, Golden Hospital, Pingtung 90049, Taiwan; ^4^Department of Biotechnology and Food Technology, College of Engineering, Southern Taiwan University of Science and Technology, Tainan 710301, Taiwan; ^5^Department of Cell Biology and Anatomy, College of Medicine, National Cheng Kung University, Tainan 70101, Taiwan; ^6^Department of Medical Research, China Medical University Hospital, China Medical University, Taichung 40402, Taiwan; ^7^Department of Nursing, Shu-Zen Junior College of Medicine and Management, Kaohsiung 82144, Taiwan

## Abstract

Since a portion of patients with nasopharyngeal carcinoma (NPC) do not benefit much from current standard treatments, it is still needed to discover new therapeutic drugs to improve the prognosis of the patients. Considering that Chinese traditional medicine plays a role in inhibiting tumor progression, in this study, we aimed to investigate whether a Chinese herbal formula, Qing Yan Li Ge Tang (QYLGT), has the anticancer activity in NPC cells and explore the underlying mechanism as well. MTT assay, colony formation assay, immunoblotting assay, and DNA laddering assay were performed to assess cell viability, cell colony formation, protein expression, and DNA fragmentation, respectively. Results show that QYLGT was able to inhibit the cell viability and decrease colony formation ability in NPC cells. QYLGT could also increase the formation of intracellular vacuoles and induce the autophagy-related protein expressions, including Atg3, Atg6, and Atg12-Atg5 conjugate in NPC cells. Treatment with an autophagy inhibitor, 3-methyladenine, could significantly recover QYLGT-inhibited cell viability of NPC cells. In addition, QYLGT did not significantly induce apoptosis in NPC cells. We also found that QYLGT had the ability to activate phosphoinositide 3-kinase (PI3K)/Akt/mammalian target of the rapamycin (mTOR) pathway. Treatment with PI3K inhibitors, LY294002 and wortmannin, or mTOR inhibitors, rapamycin and Torin 1, could not only recover QYLGT-inhibited cell viability of NPC cells but also inhibit Atg3 expression. Taken together, our results demonstrated that QYLGT could induce autophagic cell death in NPC cells through the PI3K/Akt/mTOR pathway.

## 1. Introduction

Nasopharyngeal carcinoma (NPC) is a malignant tumor with high metastatic features originating from the nasopharynx. The incidence rate of NPC is generally low (under 1 per 100,000 persons per year) around the world [1]. However, in some distinct areas in Southern China, such as Guangdong and Hong Kong, the incidence rate is rather high about 20–30 per 100,000 persons per year [2]. Although radiotherapy alone or combination with chemotherapy has become the standard treatment for patients with early stage or locally advanced NPC which contribute a 5-year survival rate of about 85–90% [3–5], a small portion of NPC patients have no beneficial effects from the treatments. Therefore, it is important and critical to discover new therapeutic strategies or drugs to improve the prognosis of NPC patients.

Several studies have shown that Chinese herbal medicine plays an important role in the prevention and treatment of cancer. For instance, the Chinese herbal formulas, such as Zeng-Sheng-Ping and Yangyinjiedu, have been shown to inhibit tumor progression in animal models [6, 7]. The extracts of traditional Chinese medicine are also reported to have the antitumor ability. For instance, some natural products and curcumin, an extract of *Curcuma longa*, are able to induce apoptosis in various cancer cell lines [8–10] and also inhibit tumor growth [11]. In addition, EFLA400, a *Panax ginseng* extract, can significantly reduce the tumor incidence induced by carcinogens [12]. Qing Yan Li Ge Tang (QYLGT), a Chinese herbal formula, is widely used in clinical applications to the patients with acute upper respiratory infection, allergic rhinitis, cough, or chronic pharyngitis; however, it is still unknown whether it has antitumor effects.

Autophagy is a regulated self-digestion mechanism, which is important in removing misfolded, aggregated proteins, and damaged organelles in cells [13]. The process of autophagy involves the formation of autophagosomes to engulf the cytoplasmic contents, which will be degraded when the autophagosomes fuse with lysosomes [13]. It is known that the process of autophagosome formation is regulated by Atg genes. For instance, Atg6, Atg3, and Atg12-Atg5 conjugate are involved in regulating the initiation, elongation, and maturation of autophagosome, respectively [14–16]. Additionally, autophagy can be induced by multiple stresses, including starvation, DNA damage, hypoxia, or cisplatin, a first line anticancer drug [17–20]. In these stresses, autophagy could be induced and may play different roles in promoting cell survival or death. For instance, autophagy can be induced by starvation condition, which is able to promote cell survival [21]. However, under the treatment of a traditional herbal medicine, Samsoeum, autophagy is involved in promoting cell death [22]. Currently, several Chinese herbal medicines and natural compounds have been shown to induce apoptosis and/or autophagic cell death in cancer cells [22–26].

In this study, we investigated whether QYLGT has anticancer activity in NPC cells and also explored the underlying mechanism. We found that QYLGT could inhibit the proliferation of NPC cells by the induction of autophagic cell death. In addition, the phosphatidylinositol 3-kinase (PI3K)/Akt/mammalian target of rapamycin (mTOR) pathway is involved in mediating the autophagic cell death induced by QYLGT. These results suggest that QYLGT has the anticancer potential, which may contribute to future clinical application on NPC therapy.

## 2. Materials and Methods

### 2.1. Cell Culture and Drug Treatment

Two NPC cell lines, HONE-1 and NPC-TW01, were obtained from Dr. Chin-Hwa Tsai (National Taiwan University, Taiwan, R.O.C) and cultured in the RPMI-1640 medium with 10% fetal bovine serum (FBS) (Hyclone, UT, USA) with 5% CO_2_ at 37°C. In order to explore whether the cellular signaling pathways, including p38 mitogen-activated protein kinase (MAPK), mitogen-activated protein kinase kinase (MEK)/extracellular signal regulated kinase (ERK), c-Jun N-terminal kinase (JNK), PI3K/Akt, and mTOR, are involved in QYLGT-induced inhibition of cell viability, their specific inhibitors were used, respectively, based on the following conditions: to inhibit the p38 MAPK pathway, cells were treated with 5 *μ*M SB203580 (Sigma, MO, USA) for 48 h; to inhibit the MEK/ERK pathway, cells were treated with 0.5 *μ*M PD184352 (Enzo Life Sciences, NY, USA) for 48 h; to inhibit the JNK pathway, cells were treated with 5 *μ*M SP600125 (Sigma, MO, USA) for 48 h; to inhibit the PI3K/Akt pathway, cells were treated with 5 *μ*M LY294002 (Sigma, MO, USA) or 2 *μ*M wortmannin (Sigma, MO, USA) for 48 h; and to inhibit the mTOR pathway, cells were treated with 0.2 *μ*M rapamycin (Sigma, MO, USA) or 1 *μ*M Torin 1 (R&D Systems, MN, USA) for 48 h. In order to investigate whether QYLGT inhibits cell viability of NPC cells through induction of autophagy, cells were treated with 10 *μ*M autophagy inhibitor, 3-methyladenine (Sigma, MO, USA), for 48 h.

### 2.2. Preparation of QYLGT

QYLGT, a commercial Chinese herbal formula purchased from Ko Da Pharmaceutical Co., Ltd. (Taoyuan, Taiwan, R.O.C), is composed by ten herbs, including Radix Scrophulariae, Rhizoma Cimicifugae, Radix Platycodi, Radix Glycyrrhizae Preparata, Poria, Rhizoma Coptidis, Radix Scutellariae, Fructus Arctii, Radix Saposhnikoviae, and Radix Paeoniae Alba. Five grams of the QYLGT were mixed with 50 ml DMSO and shaked at room temperature (RT) at 100 rpm for 24 h. Then, the mixture was centrifuged at RT for 10 min at 3,000 rpm. The supernatant was collected and then dried into powder by freeze drying. Five grams of the powder were dissolved in 50 ml PBS and sterilized by autoclaving to make 100 mg/ml stock solution, and the solution was stored at 4°C.

### 2.3. MTT Assay

Cell viability was measured by using MTT assay [27]. Cells (3 × 10^5^/well) were seeded into a 6-well plate. After reaching about 80% confluence, cells were treated with QYLGT and/or signaling pathway inhibitors. At indicated time points, the supernatants were removed, and then, 2 ml of MTT (0.5 mg/ml in PBS) was added to each well. After 4 h, the supernatants were removed, and then, 1 ml of dimethyl sulfoxide (DMSO) was added to each well to dissolve the crystals. Then, we took 100 *μ*l DMSO lysate from each well of 6-well plates and transferred the lysates into a 96-well plate. The optical density values were measured at 570 nm by an ELISA reader (BMG LABTECH, Ortenberg, Germany). Each assay was carried out in triplicate, and the whole set of experiments was performed three times independently.

### 2.4. Colony Formation Assay

HONE-1 and NPC-TW01 cells were, respectively, treated with 65 and 430 *μ*g/ml QYLGT for 48 h. Then, the cells were collected and seeded in 6-well plates at a density of 100 cells per well. The cells were incubated in RPMI-1640 containing 5% FBS at 37°C for 12 days. Then, the cells were fixed with methanol and stained with 0.1% crystal violet. The number of visible colonies was obtained by manually counting. Each assay was carried out in triplicate, and the whole set of experiments was performed three times independently.

### 2.5. Immunoblotting Assay

Protein extraction and the immunoblotting assay were performed as described previously [28]. Antibodies for detecting Atg3, Atg6, Atg12-Atg5 conjugate, cleaved caspase-3, PARP, and Akt phosphorylated at Ser473, total Akt, mTOR phosphorylated at Ser2448, and total mTOR were purchased from Cell Signaling Technology (Cell Signaling Technology, MA, USA). The anti-*β*-actin antibody was purchased from Chemicon (Chemicon, MA, USA). Representative results from at least two independent experiments are shown.

### 2.6. DNA Laddering Assay

Internucleosomal DNA fragmentation is a feature of apoptosis, which can be detected by using DNA laddering assay [29]. In this study, the assay was performed by using a DNA laddering kit (Cayman Chemical, MI, USA) according to the manufacturer's instruction. Representative results from at least two independent experiments are shown.

### 2.7. Statistical Analysis

Data were expressed as mean ± SEM and analyzed by using SPSS statistical software (version 17.0). Statistically significant differences between groups were determined by one-way ANOVA and followed by the least significant difference (LSD) test. *P* < 0.05 was considered statistically significant.

## 3. Results

### 3.1. QYLGT Inhibits Cell Viability of NPC Cells

**Figure 1:**
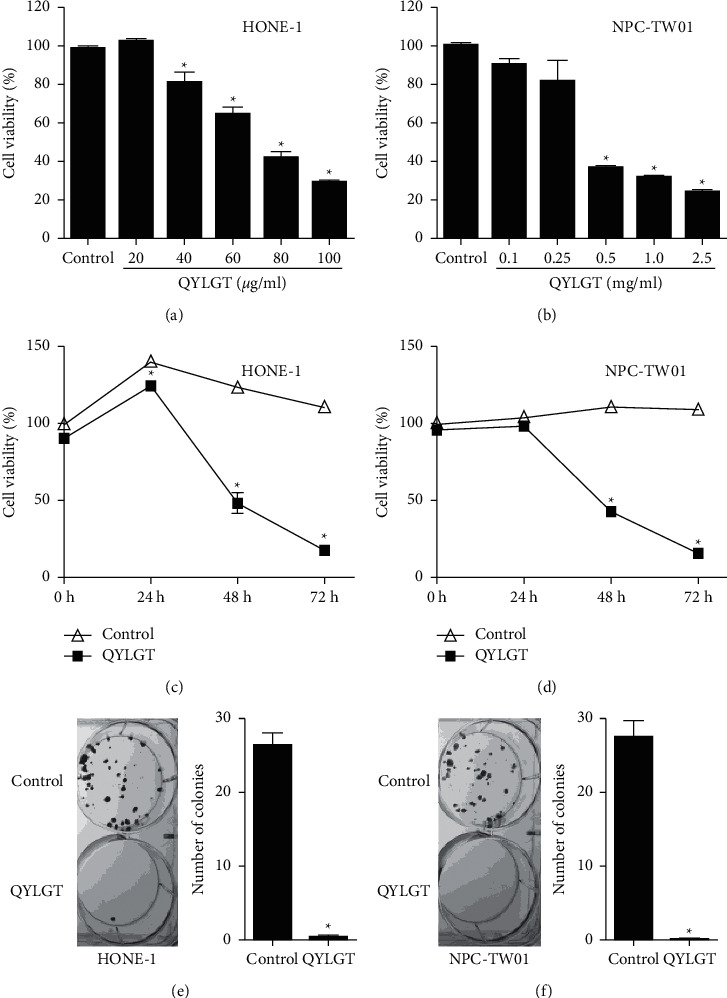
QYLGT inhibits cell viability of NPC cells. (a) HONE-1 and (b) NPC-TW01 cells were treated without (control) or with various concentrations of QYLGT for 48 h, and cell viability was measured by MTT assay. (c) HONE-1 and (d) NPC-TW01 cells were treated without (control) or treated with 65 and 430 *μ*g/ml QYLGT for 0, 24, 48, and 72 h, respectively, and cell viability was measured by MTT assay. (e) HONE-1 and (f) NPC-TW01 cells were treated without (control) or treated with 65 and 430 *μ*g/ml QYLGT, respectively, for 48 h, and clone formation ability was measured by colony formation assay. ^*∗*^*P* < 0.05 vs. control.

HONE-1 and NPC-TW01 NPC cells were treated with different doses of QYLGT (20–100 *μ*g/ml to HONE-1 cells and 0.1–2.5 mg/ml to NPC-TW01 cells, respectively), and then, cell viabilities were measured by MTT assay. We found that 40–200 *μ*g/ml QYLGT could significantly inhibit HONE-1 cell viability (Figure 1(a)). In Figure 1(b), 0.1–2.5 mg/ml QYLGT could also significantly inhibit NPC-TW01 cell viability. The IC50 values of QYLGT for HONE-1 and NPC-TW01 were 65 *μ*g/ml and 430 *μ*g/ml, respectively. We used IC50 of QYLGT as a standard concentration to further study the time-course effect on NPC cells, which were treated with IC50 of QYLGT for 0, 24, 48, and 72 h, respectively. Compared with the control, 65 *μ*g/ml QYLGT was able to obviously inhibit HONE-1 cell viability at 24, 48, and 72 h (Figure 1(c)) and 430 *μ*g/ml QYLGT also significantly inhibits NPC-TW01 cell viability at 48 and 72 h (Figure 1(d)), respectively. Next, we used colony formation assay to confirm the inhibitory effect of QYLGT on cell viability of NPC cells. Results in Figures 1(e) and 1(f) show that the clonogenic growth capacities of both NPC cells were significantly suppressed by QYLGT.

### 3.2. QYLGT Inhibits Cell Viability of NPC Cells through Induction of Autophagy

**Figure 2:**
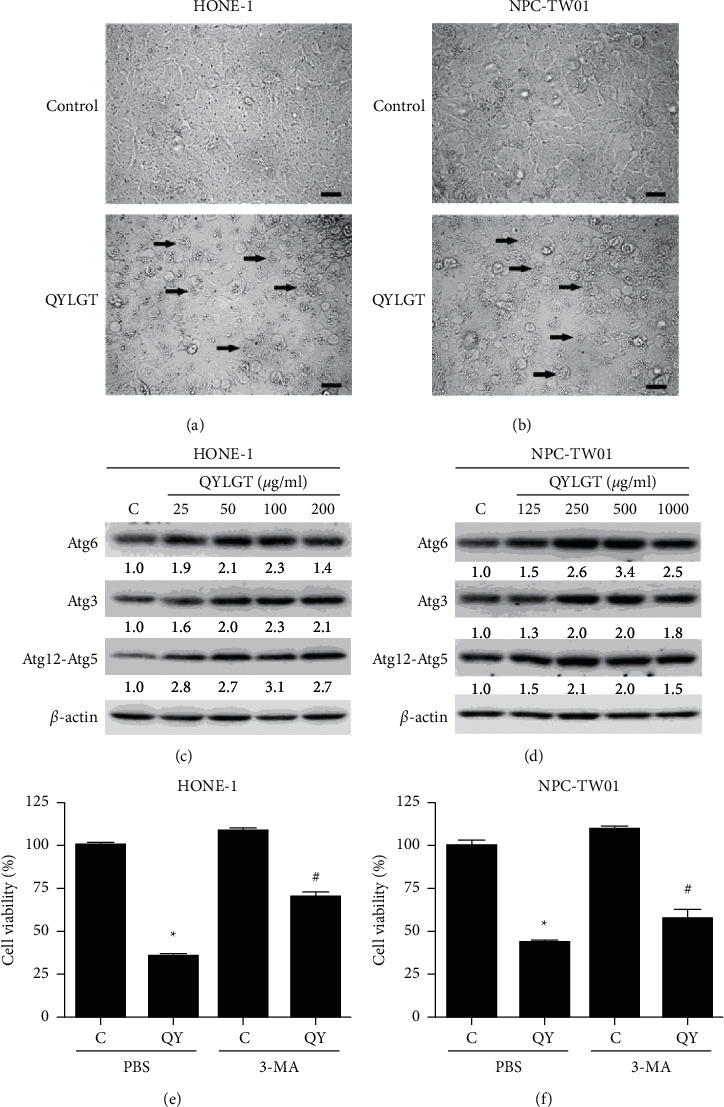
QYLGT inhibits cell viability of NPC cells through induction of autophagy. (a) HONE-1 and (b) NPC-TW01 cells were treated without (control) or with 65 and 430 *μ*g/ml QYLGT, respectively. After 48 h, the cell morphology was observed by using phase-contrast microscopy (scale bar: 100 *μ*m; arrow heads: autophagic vacuoles). (c) HONE-1 and (d) NPC-TW01 cells were treated without (C = control) or with various concentrations of QYLGT. Protein expressions of Atg6, Atg3, Atg12-Atg5, and *β*-actin were examined using an immunoblot assay. The relative expression levels of individual proteins were quantified by normalization with their corresponding *β*-actin, and the expression level in the control group was set as 1.0. (e) HONE-1 and (f) NPC-TW01 cells were treated without (C = control) or with QYLGT (QY) and concurrently treated with the solvent control PBS or with an autophagy inhibitor, 3-methyladenine (3-MA), respectively. The cell viability was measured by MTT assay. ^*∗*^*P* < 0.05 vs. control + PBS group. ^#^*P* < 0.05 vs. the QYLGT + PBS group.

Cell morphology between control and QYLGT-treated cells was then examined. We found that, compared to control, more vacuoles were formed in the QYLGT-treated NPC cells (Figures 2(a) and 2(b)), suggesting that autophagy is induced by QYLGT. Several autophagy markers, including Atg6, Atg3, and Atg12-Atg5 conjugate, have been shown to play important roles in regulating the initiation, elongation, and maturation of autophagosome, respectively [14–16]. Thus, we further confirmed whether QYLGT could trigger autophagy in NPC cells. NPC cells were treated with different doses of QYLGT, and the autophagy markers were detected by an immunoblotting assay. Results in Figures 2(c) and 2(d) show that QYLGT could upregulate the expression of Atg6, Atg3, and Atg12-Atg5 conjugated in both NPC cell lines, suggesting that autophagy is induced by QYLGT in NPC cells. We wondered whether autophagy is involved in QYLGT-inhibited viability of NPC cells. We further used the autophagy inhibitor, 3-methyladenine, which can inhibit autophagosome formation, to confirm QYLGT did induce autophagic phenomenon in NPC cells. Data in Figures 2(e) and 2(f) show that 3-methyladenine was able to recover QYLGT-induced cell viability loss in NPC cells, indicating autophagy in NPC cells was activated by QYLGT.

### 3.3. QYLGT Does Not Significantly Induce Apoptotic Cell Death in NPC Cells

**Figure 3:**
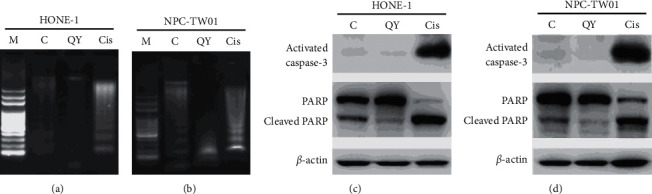
QYLGT does not significantly induce apoptotic cell death in NPC cells. HONE-1 and NPC-TW01 cells were treated without (C=control) or treated with QYLGT (QY) and cisplatin (Cis), respectively. DNA fragmentation was detected by using DNA laddering assay ((a) for HONE-1 cells and (b) for NPC-TW01 cells). Protein expressions of activated caspase-3, PARP, cleaved PARP, and β-actin were examined by immunoblot assay ((c) for HONE-1 cells and (d) for NPC-TW01 cells).

We wondered whether QYLGT would induce apoptotic cell death in NPC cells. Thus, apoptotic-related features, including DNA fragmentation, caspase-3 activation, and cleavage of PARP [30], were examined by DNA laddering assay and immunoblotting assay, respectively. The data from DNA laddering assay (Figures 3(a) and 3(b)) and immunoblotting assay (Figures 3(c) and 3(d)) show that, in both NPC cell lines, the control groups slightly showed DNA ladder formation and the expression of cleaved caspase-3 and PARP proteins, which could be due to the serum starvation. In contrast, the QYLGT did not significantly induce DNA ladder formation (Figures 3(a) and 3(b)) and the expression of cleaved caspase-3 and PARP proteins (Figures 3(c) and 3(d)). In addition, acting as a positive control, the cisplatin did stimulate apoptotic features in NPC cells (Figures 3(a)–3(d)).

### 3.4. QYLGT Activates the PI3K/Akt/mTOR Pathway for Autophagic Cell Death in NPC Cells

**Figure 4:**
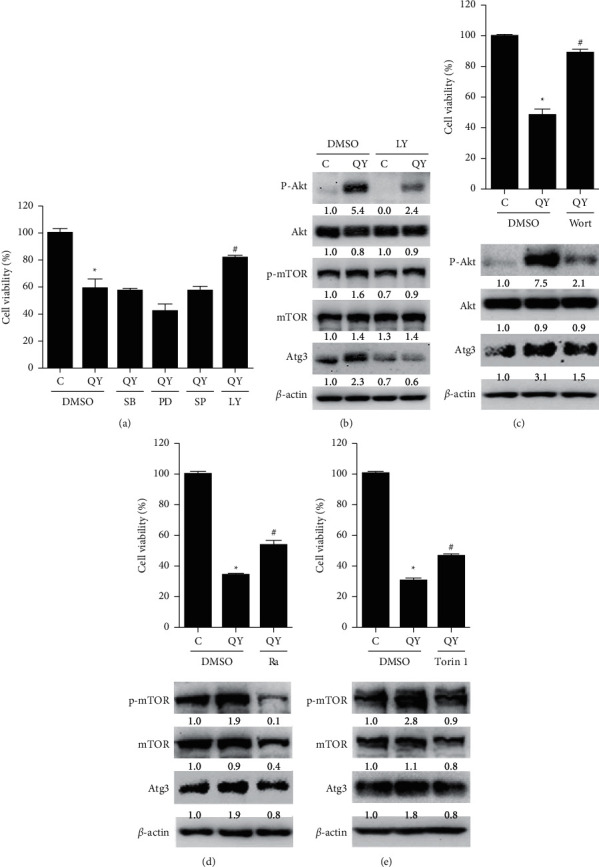
QYLGT induces activation of the PI3K/Akt/mTOR pathway, which is required for autophagic cell death in NPC cells. (a) HONE-1 cells were treated without (C = control) or with QYLGT (QY) and concurrently treated with solvent control DMSO, a p38 MAPK inhibitor SB202190 (SB), an ERK inhibitor PD184352 (PD), a JNK inhibitor SP600125 (SP), or a PI3K/Akt inhibitor LY294002 (LY), respectively. The cell viability was measured by MTT assay. (b) HONE-1 cells were treated without (C=control) or with QYLGT (QY) and concurrently treated with solvent control DMSO or LY294002 (LY). Protein expressions of phosphorylated Akt (p-Akt), total Akt (Akt), phosphorylated mTOR (p-mTOR), total mTOR (mTOR), Atg3, and *β*-actin were examined by an immunoblot assay (c) HONE-1 cells were treated without (C=control) or with QYLGT (QY) and concurrently treated with solvent control DMSO or wortmannin (Wort). Upper panel: cell viability was measured by MTT assay. Lower panel: protein expressions of phosphorylated Akt (p-Akt), total Akt (Akt), Atg3, and *β*-actin were examined by immunoblot assay. (d) HONE-1 cells were treated without (C=control) or with QYLGT (QY) and concurrently treated with solvent control DMSO or rapamycin (Ra). Upper panel: cell viability was measured by MTT assay. Lower panel: protein expressions of phosphorylated mTOR (p-mTOR), total mTOR (mTOR), Atg3, and *β*-actin were examined by immunoblot assay. (e) HONE-1 cells were treated without (C=control) or with QYLGT (QY) and concurrently treated with solvent control DMSO or Torin 1. Upper panel: cell viability was measured by MTT assay. Lower panel: protein expressions of phosphorylated mTOR (p-mTOR), total mTOR (mTOR), Atg3, and *β*-actin were examined by an immunoblot assay. The relative expression levels of individual proteins were quantified by normalization with their corresponding *β*-actin, and the expression level in the control group was set as 1.0. ^*∗*^*P* < 0.05 vs. the control + DMSO group. ^#^*P* < 0.05 vs. the QYLGT + DMSO group.

Since several cellular signaling pathways, including p38 MAPK, MEK/ERK, JNK, and PI3K/Akt/mTOR, are associated with autophagy and/or apoptosis inductions [31–34], we investigated which pathway is the major one required for QYLGT-induced autophagic cell death in NPC cells. The specific inhibitors of p38, MEK/ERK, JNK, and PI3K/Akt, including SB202190, PD184352, SP600125, and LY294002, respectively, were used. We found that the PI3K/Akt inhibitor, LY294002, could significantly inhibit QYLGT-induced cell death (Figure 4(a)). In addition, LY294002 not only suppressed the expression of PI3K and mTOR, a PI3K downstream molecule (Figure 4(b)), but also reduced the expression of Atg3, the autophagy marker (Figure 4(b)). To further confirm the role of PI3K/Akt inhibiting QYLGT-induced cell death, NPC cells were treated with another PI3K/Akt specific inhibitor, wortmannin.  Figure 4(c) shows that wortmannin suppressed QYLGT-induced PI3K activation and Atg3 expression plus QYLGT-induced cell death, respectively. Furthermore, we also treated NPC cells with mTOR inhibitors, rapamycin and Torin 1. We found that rapamycin (Figure 4(d)) and Torin1 (Figure 4(e)) could inhibit QYLGT-induced mTOR activation and Atg3 expression plus QYLGT-induced cell death. These results indicate that the PI3K/Akt/mTOR pathway is important for QYLGT-induced autophagic cell death in NPC cells.

## 4. Discussion

Considering Chinese herbal medicines have anticancer activities in various types of cancer cells [22–26], this study aimed to test whether a Chinese herbal formula, QYLGT, has the anticancer activity in NPC cells. Our results indicate that QYLGT can significantly inhibit cell viability of HONE-1 cells (Figure 1(a)) and NPC-TW01 cells (Figure 1(b)). We also found that the IC50 of QYLGT in HONE-1 cell is 65 *μ*g/ml, while the IC50 of QYLGT in NPC-TW01 cell is 430 *μ*g/ml, indicating that HONE-1 cell is more sensitive to QYLGT. A previous study shows that under cisplatin treatment, compared with the NPC-TW01 cells, HONE-1 cells are more sensitive to cisplatin due to HONE-1 cells express higher levels of MAD2 protein [35]. Therefore, it is highly possible that MAD2 protein expression between NPC-TW01 and HONE-1 cells could be different related to sensitivity to QYLGT treatment.

Our results show that the PI3K/Akt/mTOR pathway is important for QYLGT-induced inhibition of NPC cell viability (Figure 4). However, it is still unclear how QYLGT activates the pathway. *Coptis chinensis* is a composition of the QYLGT, and its active constituent, berberine, has been shown to induce the generation of reactive oxygen species (ROS) [36], which is reported to have the ability to activate PI3K/Akt [37]. On the other hand, another QYLGT component, Fructus Arctii, is able to activate AMP-activated protein kinase (AMPK) [38]. In fact, one study has illustrated that activation of AMPK is also associated with PI3K/Akt activation [39]. According to these clues, we postulate that QYLGT may induce the activation of PI3K/Akt through generation of ROS and/or activation of AMPK.

It is well known that the PI3K/Akt/mTOR pathway is involved in regulating autophagy in different situations. For example, some growth factors are able to activate PI3K/Akt/mTOR to suppress autophagy [40]. In addition, certain signaling pathway could be inhibited by certain chemical medicine, which results in inducing autophagy [41]. Our study found that the PI3K/Akt/mTOR pathway is activated by QYLGT, and this pathway is also involved in mediating the autophagic cell death (Figures 4(b)–4(d)). How does QYLGT-activated PI3K/Akt/mTOR promotes autophagy in NPC cells remains unclear. A previous study shows that PI3K/Akt can promote the expression of a translation factor, eukaryotic translation initiation factor (eIF) 5A [42], which is known to further mediate Atg3 expression [43]. In addition, eIF4, a downstream effector of mTOR, has been demonstrated to interact with an RGG motif protein, Psp2, to upregulate the protein expression of Atg1 and Atg13 [44]. According to these references, we speculate that QYLGT-activated PI3K/Akt/mTOR pathway may regulate autophagy via these mechanisms in NPC cells, which will be worth to further investigate the detail mechanism related to signal pathways.

Cisplatin is a clinical drug for NPC treatment, and it can be combined with radiotherapy to improve the prognosis of patients with NPC [45, 46]. However, recent studies indicate that some NPC cells have developed resistance to cisplatin [47, 48], which may explain why some of the patients did not benefit much from the treatment. Also, several studies have shown that Chinese herbal medicines not only have anticancer activities but also enhance the therapeutic effect of cisplatin [49, 50]. Our study found that QYLGT could induce autophagic cell death in NPC cells, suggesting that QYLGT could be applied to combine with cisplatin to treat NPC to enhance the therapeutic effects of cisplatin, which may contribute to a better prognosis for patients with NPC.

## 5. Conclusion

In conclusion, the in vitro study demonstrated that QYLGT has the ability to induce autophagic cell death in NPC cells via the PI3K/Akt/mTOR pathway, which may provide the evidences for future clinical application of NPC treatment. In the future, the related in vivo experiment will be carried out to further confirm the in vitro findings in this study.

## Data Availability

The data used and/or analyzed in the present study are available from the corresponding authors upon request.
